# NH_2_-MIL-125-Derived N-Doped TiO_2_@C Visible Light Catalyst for Wastewater Treatment

**DOI:** 10.3390/polym16020186

**Published:** 2024-01-08

**Authors:** Wenbin Wang, Wei Qiang, Chuntao Chen, Dongping Sun

**Affiliations:** 1 Institute of Chemicobiology and Functional Materials, School of Chemistry and Chemical Engineering, Nanjing University of Science and Technology, Nanjing 210094, China; 18302596016@163.com (W.W.); zhnlg@sina.cn (W.Q.); chchunt@njust.edu.cn (C.C.); 2 Guizhou Panjiang Civil Explosion Co., Ltd., Guiyang 551404, China

**Keywords:** titanium dioxide, NH_2_-MIL-125, N-doped TiO_2_@C, visible light photocatalysis, wastewater pollutants

## Abstract

The utilization of titanium dioxide (TiO_2_) as a photocatalyst for the treatment of wastewater has attracted significant attention in the environmental field. Herein, we prepared an NH_2_-MIL-125-derived N-doped TiO_2_@C Visible Light Catalyst through an in situ calcination method. The nitrogen element in the organic connector was released through calcination, simultaneously doping into the sample, thereby enhancing its spectral response to cover the visible region. The as-prepared N-doped TiO_2_@C catalyst exhibited a preserved cage structure even after calcination, thereby alleviating the optical shielding effect and further augmenting its photocatalytic performance by increasing the reaction sites between the catalyst and pollutants. The calcination time of the N-doped TiO_2_@C-450 °C catalyst was optimized to achieve a balance between the TiO_2_ content and nitrogen doping level, ensuring efficient degradation rates for basic fuchsin (99.7%), Rhodamine B (89.9%) and tetracycline hydrochloride (93%) within 90 min. Thus, this study presents a feasible strategy for the efficient degradation of pollutants under visible light.

## 1. Introduction

The 21st century is all about global economic development, but there are some problems that we cannot ignore. One of the common concerns is how to maintain or even improve the quality of water. According to the reports of the United Nations Educational, Scientific and Cultural Organization in the World 2021 Water Resources Development Report, as our society continues to grow, we use more and more fresh water in industry, agriculture and urban life. Since 1980, its usage has been increasing by approximately 1% every year, which exacerbates the issue of water pollution [1]. Around 80% of wastewater is being discharged into the environment without any treatment. This leads to water pollution that causes approximately 2 million deaths worldwide annually and leaves many others suffering from chronic diseases [2]. Therefore, we must consider how we can upgrade our existing methods of treating water to meet the growing demand for wastewater disposal.

The composition of wastewater usually includes organic dye pollution and antibiotic pollution, which are typical troublemakers [3]. Organic dye pollution is mainly caused by industries such as textiles, cosmetics, food and paper. It has a strong color, high toxicity to living things, exhibits excellent resistance to oxidation and is tough to break down naturally. All these factors make it a big threat to our ecosystem [4,5]. Basic fuchsin and Rhodamine B are representative organic dyes. The molecular formula of basic fuchsin is C_20_H_20_ClN_3_. Long-term exposure to basic fuchsin can cause skin cells to become cancerous because its carcinogenic triphenylmethane structure makes it a cationic dye, which is more toxic than anionic dyes. Rhodamine B, with a molecular formula of C_28_H_31_ClN_2_O_3_, is an alkaline industrial dye that poses direct harm to human health. It belongs to the third class of carcinogens in the list published by the International Agency for Research on Cancer of the World Health Organization (WHO). Antibiotic contamination usually originates from livestock and pharmaceutical industries [6]. It is difficult for animals and humans to fully absorb or transform tetracycline hydrochloride, resulting in over 50% of it being excreted through feces and urine into the environment. As a result, tetracycline hydrochloride can be detected in surface water, groundwater, sediment, soil and even drinking water. This is the main reason for the increase in bacterial resistance among humans. Growing drug resistance leads to higher healthcare costs and makes diseases more challenging to treat. Therefore, the degradation of basic fuchsin, Rhodamine B and tetracycline hydrochloride was studied in this paper’s experiments.

Currently, various technologies are being widely studied to address the challenge of water pollution, including biological methods, adsorption methods, membrane separation and photocatalysis. The biological method decomposes pollutants through the adsorption capacity and metabolic processes of bacteria, fungi, yeast, actinomycetes, algae and other microorganisms. This degradation process is simple and stable but requires strict environmental conditions. If researchers operate improperly or without cautionary measures in place, it can easily lead to secondary pollution; therefore, implementing this method on a large scale is not practical [7]. The adsorption method involves the use of physicochemical interactions between the adsorbent material and the pollutant, resulting in the adsorption of pollutants onto the material. Hani et al. reported the processing of MOFs via the three-dimensional (3D) printing of cellulose MOFs as adsorbents and catalysts for water treatment, and the materials offered the complete (>99%) removal of organic dyes within 10 min toward anionic dyes, e.g., methyl blue (MeB). The reaction conditions are mild and fast, and the adsorbent materials can be reused. However, changes in reaction conditions make it easier for them to be released again [8]. Membrane separation is a novel technology that utilizes selective permeation of membranes to separate pollutants from wastewater, including reverse osmosis, microfiltration, ultrafiltration, nanofiltration and other specific methods. It offers simple and pollution-free operation. However, when the membrane becomes blocked by pollutants, restoring it to its original state using existing cleaning technologies can be challenging. As a result, the treatment efficiency of the membrane continuously decreases, and reusing it becomes difficult. In practical applications, membrane separation is often combined with other techniques to enhance utilization [9]. Therefore, photocatalysis, which uses solar excitation to produce strong oxidizing substances, is an effective and promising means for purifying phenolic water. The photocatalytic performance of TiO_2_ is affected by the large band gap (3.2 eV of anatase), which can only absorb ultraviolet light, accounting for about 5% of the solar spectrum [10,11,12]. In addition, the rapid recombination of photogenerated electron–hole pairs in the catalytic process also reduces the photoutilization rate of TiO_2_, resulting in a decrease in the degradation efficiency of organic pollutants [7].

Anatase TiO_2_ belongs to the tetragonal system. The octahedral unit TiO_6_^2−^ has four edges. The band gap of anatase TiO_2_ is 3.2 eV, which is higher than that of rutile TiO_2_ (3.0 eV). Although this means that anatase TiO_2_ has a narrower range of light absorption, the difference in the conduction band and valence band positions gives the electron–hole pair of anatase TiO_2_ a greater positive or negative potential, thus possessing a stronger oxidation capacity. After performing theoretical calculations, Georg et al. [13] demonstrated that the surface of anatase TiO_2_ demonstrates a high adsorption capacity for H_2_O, O_2_ and OH^−^, leading to the increased production of active free radicals during catalytic reactions and enhanced photocatalytic activity. During the crystallization process, anatase TiO_2_ tends to form small-sized particles with a large specific surface area [14], which is also beneficial for the photocatalytic reaction [15].

Metal–organic frameworks (MOFs) are materials that consist of a self-assembled metal–organic skeleton formed by clusters of metal ions and organic ligands [16]. NH_2_-MIL-125 is a type of MOF that has a large specific surface area and pore volume, and it can facilitate transmission and diffusion and expose as many active sites as possible [17]. The porous structure of NH_2_-MIL-125 allows for a shorter distance for charge carrier transfer, effectively separating photogenerated electron pairs [18]. Furthermore, the large, interconnected 3D open cavities in NH_2_-MIL-125 facilitate easy penetration of light, effectively reducing light shielding [19]. The catalyst prepared using NH_2_-MIL-125 as the template combines TiO_2_ with a carbon matrix, which not only maintains the morphological advantage of the MOF but also limits the aggregation of TiO_2_ nanocrystals and improves the electrical conductivity of the TiO_2_@C composite [20,21,22,23,24].

In this work, NH_2_-MIL-125 was used as a template to synthesize N-doped TiO_2_@C composite catalyst with high photocatalytic efficiency via calcination. TiO_2_ grew in situ with titanium elements in NH_2_-MIL-125 as the core, and the carbon skeleton of NH_2_-MIL-125 as a template still existed after calcination. The structure of N/TiO_2_@C catalytic materials was characterized using XRD, SEM, TEM, XPS and other testing methods. The natural and photocatalytic properties of N-doped TiO2@C composites were evaluated.

## 2. Materials and Methods

### 2.1. Materials

2-amino-terephthalic acid (99%) was bought from Beijing Bailingwei Technology Co., Ltd., Beijing, China. Dimethylformamide (99.9%) was bought from Aladdin Chemical Co., Ltd., Shanghai, China. Methanol (99.9%) was bought from Jinan Century Tongda Co., Ltd., Jinan, Chian. Isopropyl titanate (95%) was obtained from Syntechem Co., Ltd., Guangzhou, China.

### 2.2. Preparation of NH_2_-MIL-125

NH_2_-MIL-125 was prepared through a hydrothermal reaction. An amount of 1 g of 2-amino-terephthalic acid was added to a mixture of 18 mL of dimethylformamide and 2 mL of methanol. The mixture was stirred and subjected to ultrasonication for 5 min, followed by the addition of 1.2 mL of isopropyl titanate. After stirring and ultrasonication for 5 min, it was transferred to a Teflon autoclave with a volume capacity of 50 mL for heat treatment at 150 °C for 15 h. The resulting yellow powder was collected, centrifuged, washed three times each with dimethylformamide/ethanol and subsequently dried under vacuum conditions at 50 °C for 20 h.

### 2.3. Preparation of N-Doped TiO_2_@C Nanomaterials

N-doped TiO_2_@C was prepared via the one-step pyrolysis method. A porcelain boat containing 500 mg of NH_2_-MIL-125 powder was positioned at the center of a high-temperature tubular furnace for air-based heat treatment. The tubular furnace operated with a heating rate set to 2 °C·min^−1^ within a target temperature range spanning from 200 °C to 550 °C while maintaining the desired temperature for two hours. Following the completion of the reaction, we retrieved the resulting powder.

### 2.4. Characterization

Chemical groups within samples were investigated using a Nicolet IS 20 FTIR spectrometer (Thermo Fisher, Waltham, MA, USA) in the range of 4000–500 cm^−1^. The X-ray diffraction (XRD) of samples was conducted with an X-ray diffractometer (D8 Advance Bruker, Berlin, Germany). The morphological structure of the cryogels was observed with a JSM-IT500HR scanning electron microscope (SEM) and a transmission electron microscope (TEM) (JEM-2100, JEOL, Kyoto, Japan). The Brunauer–Emmett–Teller (BET) surface area and pore size were tested via nitrogen adsorption and desorption at 77 K with an ASAP automated micromeritics system. The elemental composition of samples was measured via X-ray photoelectron spectroscopy (PHI QUANTERA II, Ulvac-Phi, Kanagawa, Japan). Thermogravimetric analysis (TGA, METTLER TOLEDO TGA/SDTA851) was carried out at a constant heating rate of 10 °C min^−1^ from 50 °C to 700 °C under a nitrogen atmosphere. The optical performance of the samples was conducted on a UV-3600 spectrophotometer (Shimadzu, Kyoto, Japan). The electrochemical tests, including the electrochemical impedance (EIS), transient photocurrent and Mott–Schottky (M–S), were pictured with an electrochemical workstation (RST5200F, Restile, Gujarat, India). The response of hydroxyl radicals was tested with an electron paramagnetic resonance (EPR) spectrometer (EMXPLUS, Bruker). The contents of C, N and H in the resultant cellulose samples were determined with a Eurovector EA 3000 elemental analyzer in CHN mode.

### 2.5. Photocatalytic Performance Measurements

Visible light irradiation (more than 400 nm) degraded the basic fuchsin, Rhodamine B and tetracycline hydrochloride, and the photocatalytic properties of various catalysts were studied. All photocatalytic experiments were repeated three times. A 300 W xenon lamp with a filter was used to simulate AM 1.5 G lighting (100 mW/cm^2^) as a light source. The circulating water flow in the reactor ensures that the photocatalytic reaction takes place at room temperature. During the degradation process, 20 mg of each photocatalyst was separately dispersed into 30 mL of basic fuchsin, Rhodamine B and tetracycline hydrochloride at a concentration of 15 mg/L. The mixture was subsequently stirred in darkness for 60 min to achieve adsorption–desorption equilibrium. During irradiation, the samples were regularly extracted and filtered using a 0.45 μm PTFE needle filter. The resulting solution was measured for absorbance at wavelengths of 546 nm, 554 nm and 370 nm utilizing an ultraviolet–visible spectrophotometer. After degradation, the catalyst underwent recovery through filtration before being washed with deionized water and ethanol. Subsequently, it was dried under vacuum conditions at a temperature of 60 °C for twenty hours. Finally, the treated catalyst could undergo multiple tests to evaluate its stability.

## 3. Results

### 3.1. Synthesis and Characterization of N-Doped TiO_2_@C

**Figure 1:**
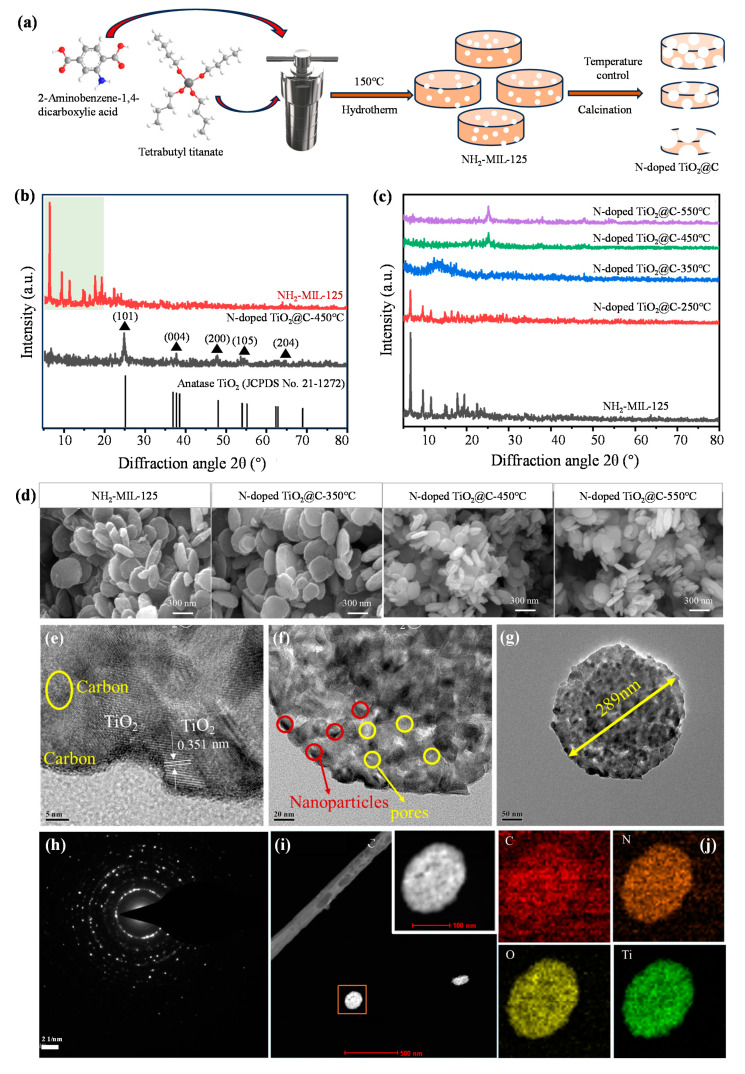
Schematic diagram and characterization of N-doped TiO_2_@C composite. (**a**) Fabrication of N-doped TiO_2_@C composite. (**b**,**c**) NH_2_-MIL-125 and N-doped TiO_2_@C composites with calcination temperatures of 250 °C, 350 °C, 450 °C and 550 °C. (**d**) SEM images of NH_2_-MIL-125 and N-doped TiO_2_@C composites with calcination temperatures of 250 °C, 350 °C, 450 °C and 550 °C. (**e**–**g**) TEM images of N-doped TiO_2_@C-450 °C. (**h**) Electron diffraction image of N-doped TiO_2_@C-450 °C. (**i**,**j**) Element mapping of N-doped TiO_2_@C-450 °C.

The overall fabrication of the N-doped TiO_2_@C composite is schematically illustrated in Figure 1a. In order to explore the crystal structure of the sample prepared in the experiment, X-ray diffraction (XRD) tests were first conducted. NH_2_-MIL-125 samples show typical diffraction peaks at 6.67°, 9.62°, 11.48°, 14.90°, 16.50°, 17.89° and 19.42°. The outcomes indicated that the synthesis of NH_2_-MIL-125 was successful and that the produced MOF exhibited acceptable crystallinity. N-doped TiO_2_@C-450 °C was obtained via the calcination of NH_2_-MIL-125, and the characteristic peak of NH_2_-MIL-125 totally vanished during the process. The XRD pattern of N-doped TiO_2_@C-450 °C was compared with the standard diffraction card of acanite TiO_2_ (JCPDSNo.21−1272). It was found that peaks of 25.06°, 38.10°, 47.89°, 53.70° and 61.46° corresponded to the (101), (004), (200), (105) and (204) planes of anatase, respectively. The results show that the pyrolysis of NH_2_-MIL-125 to produce anatase TiO_2_ was successful. In addition, the XRD characterization of N-doped TiO_2_@C was significantly different from different calcination temperatures (Figure 1b,c). The calcination temperature of N-doped TiO_2_@C-250 °C was too low, which caused insufficient pyrolysis. The characteristic peaks of NH_2_-MIL-125 can still be seen at 6.63°, 9.65° and 11.64°, but the peak intensity was reduced significantly. This may have been due to the partial disintegration and recombination of the NH_2_-MIL-125 structure. When the temperature of the calcination reached 350 °C, only a carbon sheath peaked at 13.19°, and no TiO_2_-related peak pattern was visible, indicating that the NH_2_-MIL-125 structure was totally broken down. TiO_2_ had not yet formed crystals, and the carbon element that was liberated during the calcination was doped into the compound. The distinctive peak of anatase TiO_2_ was clearly seen when the temperature hit 450 °C. Following that, the intensity of the anatase TiO_2_ characteristic peak no longer varied considerably with the rise in calcination temperature, but the crystallinity of TiO_2_ was more excellent.

From the SEM images, the prepared NH_2_-MIL-125 showed a circular flake shape with an average diameter of about 422 nm (Figure 1d). In N-doped TiO_2_@C-450 °C, the thickness of TiO_2_-like circular tablets was about 50 nm, and the diameter was about 140 nm. The sample had a thickness of around 60 nm and a diameter of about 370 nm for N-doped TiO_2_@C-350 °C. And for the N-doped TiO_2_@C-250 °C sample, its thickness was around 70 nm, and its diameter was about 450 nm. It is evident that, as the calcination temperature rose, the average particle size of the sample particles shrank to some extent while maintaining its round pill shape. This might have been because, as the calcination temperature rose, the network structure of NH_2_-MIL-125 gradually lost its capacity to crystallize, and the organic molecules in the skeleton broke down.

The shape and crystal structure of N-doped TiO_2_@C-450 °C composites were obtained via TEM. The observation of amorphous carbon shows that a large amount of TiO_2_ was distributed in the carbon matrix and finally formed the TiO_2_@C composite. The sample obtained after calcination still maintained a porous structure, as seen by the presence of TiO_2_ nanoparticles and pores (Figure 1e,f). This porous construction can increase photocatalytic efficiency by exposing as many active sites as feasible while also facilitating the transmission and diffusion of electrons during catalysis. In the adsorption experiment, more investigation into the porous structure was conducted on N/TiO_2_@C-450 °C, on which a lattice spacing of 0.351 nm was found, compatible with the lattice spacing of the crystal planes of anatase TiO_2_ (101) (Figure 1g). From TEM observations, the diameter of the N-doped TiO_2_@C-450 °C was approximately 289 nm (Figure 1g). On N-doped TiO_2_@C-450 °C, a lattice spacing of 0.351 nm was found, which was compatible with the lattice spacing of the crystal planes of anatase TiO_2_ (101) (Figure 1h). Titanium, oxygen, carbon and nitrogen were all further confirmed by the element mapping (Figure 1i,j). It is evident that, on N-doped TiO_2_@C-450 °C, the distribution of titanium, oxygen, carbon and nitrogen was quite uniform. It can be argued that, during calcination, the nitrogen element contained in the organic ligand 2-amino-terephtharic acid was released and then doped into the sample. The titanium element in NH_2_-MIL-125 was grown in situ to form TiO_2_, which was uniformly dispersed in the porous carbon skeleton, and ultimately, the nitrogen-doped TiO_2_@C was successfully formed.

**Figure 2:**
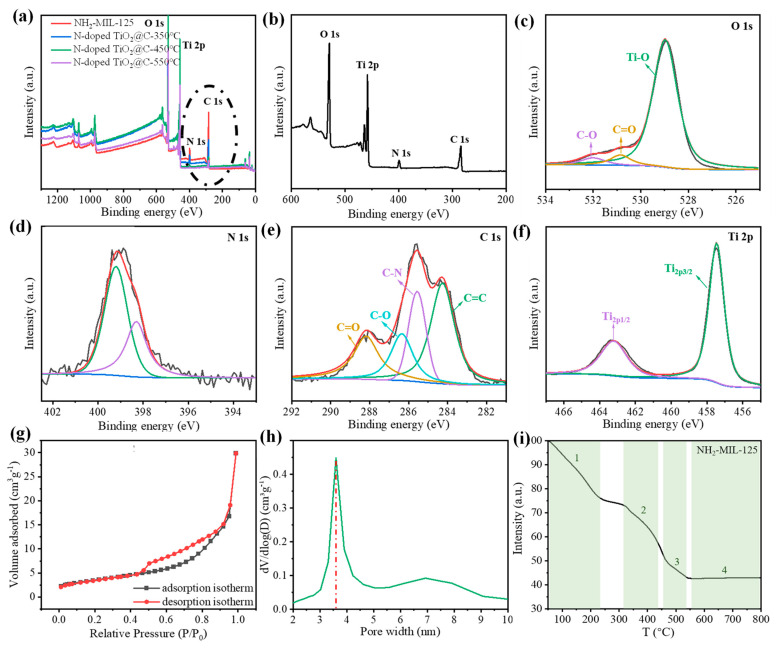
Chemical characterization of the N-doped TiO_2_@C composites. (**a**,**b**) Survey XPS spectra of NH_2_-MIL-125, N-doped TiO_2_@C-250 °C, N-doped TiO_2_@C-350 °C, N-doped TiO_2_@C-450 °C and N-doped TiO_2_@C-550 °C. High-resolution XPS spectra of (**c**) O 1s, (**d**) N 1s, (**e**) C 1s and (**f**) Ti 2p of N-doped TiO_2_@C-450 °C. (**g**) N_2_ adsorption–desorption isotherms of N-doped TiO_2_@C-450 °C. (**h**) Pore size distributions of N-doped TiO_2_@C-450 °C. (**i**) Thermogravimetric curve of NH_2_-MIL-125.

The elemental composition and valence information of N-doped TiO_2_@C were subsequently examined using X-ray photoelectron spectroscopy (XPS), which was utilized to test the material composition (Figure 2a,b). When the temperature was too high, all nitrogen was released and was no longer doped into the TiO_2_@C composite, and the peak value of carbon and nitrogen decreased with the increase in calcination temperature. When the temperature reached 550 °C, the peak of nitrogen was close to zero. However, the proper calcination temperature was crucial, as the XRD pattern analysis showed that anatase TiO_2_ was difficult to produce or had poor crystallinity at too low temperatures. A calcination temperature of 450 °C could be used as the equilibrium temperature between nitrogen doping and TiO_2_ crystallinity. Three peaks at 532.0 eV, 530.9 eV and 528.9 eV, which are associated with the C-O bond, C=O bond and Ti-O bond, respectively, can be seen in the XPS spectra of O 1s (Figure 2c). The surface hydroxyl oxygen, which is essential for the photodegradation of contaminants, was responsible for the development of the C-O bond. To fight contaminants, the hydroxyl radical OH• could be created when it interacted with photogenic holes. N 1s (Figure 2d) displayed a broad peak between 398.4 and 399.4 eV. It is consistent with other results and typical Ti-N structures in nitrogen-doped TiO_2_ [25]. The C=C peaks (Figure 2e) at 288.2 eV, 286.4 eV, 285.6 eV and 284.2 eV correspond to C=O, C-O, C-N and C=C, respectively. For pure anatase TiO_2_, Ti-O peaks were located at 458.5 eV and 464.5 eV. The Ti 2p peaks for N-doped TiO2@C-450 °C were 457.5 eV and 463.5 eV, which is a 6.0 eV energy split. The peaks of Ti 2p shown in Figure 2f centered at about 463.2 eV and 457.5 eV belonged to Ti 2p3/2 and Ti 2p1/2, respectively, which confirm the Ti^4+^ species in the form of TiO_2_ nanoparticles. The Ti 2p peak for N-doped TiO_2_@C-450 °C was slightly shifted toward a lower binding energy when compared to pure anatase TiO_2_, which was brought on by nitrogen doping altering the local chemical environment of titanium ions. The successful doping of nitrogen into TiO_2_@C, as shown by the test findings from XPS, has positive implications for reducing the band gap width of TiO_2_ and increasing its visible light activity. We also tested the relative amount of C and N in the sample with an elemental analyzer. The content of nitrogen decreased with the increase in heat treatment temperature. A ratio as high as 0.12% N/C was obtained for the heat treatment of 450 °C.

The nitrogen adsorption–desorption isotherm samples for N-doped TiO2@C-450 °C (Figure 2g) exhibited the conventional type IV isotherm and type H3 hysteresis loop. The pore size distribution was calculated using the nonlocal density function model (NLDFT) based on data from N_2_ adsorption (Figure 2h). N-doped TiO_2_@C-450 °C has a specific surface area of 63 m^2^/g, which is significantly less than that of NH_2_-MIL-125 but still significantly larger than that of other TiO_2_ catalysts. Therefore, basic fuchsin, rhodamine B and tetracycline hydrochloride can be degraded at more active sites thanks to the large specific surface area of N-doped TiO_2_@C-450 °C.

BET data need to be interpreted in combination with thermogravimetric curves (Figure 2g,h, Table 1). The specific surface area of the samples decreased, and the average pore size increased with increasing roasting temperatures, but for different reasons. The sample changed at about 200 °C as a result of the elimination of any remaining solvent. When the temperature reached 350 °C, the porous structure collapsed, and the crystal skeleton began to disintegrate, greatly reducing the specific surface area. The decomposition of the crystal skeleton and the collapse of the porous structure made the specific surface area decrease sharply. When the calcination temperature was further increased to 450 °C, the BET data changed due to the recrystallization of TiO_2_ and the formation of pyrolytic carbon substrates. However, the skeleton shrank significantly once more at 550 °C, and the specific surface area dropped to 13 m^2^/g, making it difficult for contaminants and the catalyst to make contact. Therefore, by adjusting the calcination temperature, the N-doped TiO_2_@C catalyst with the best photocatalytic effect was created at 450 °C.

### 3.2. Photocatalytic Degradation of N-Doped TiO_2_@C

**Figure 3:**
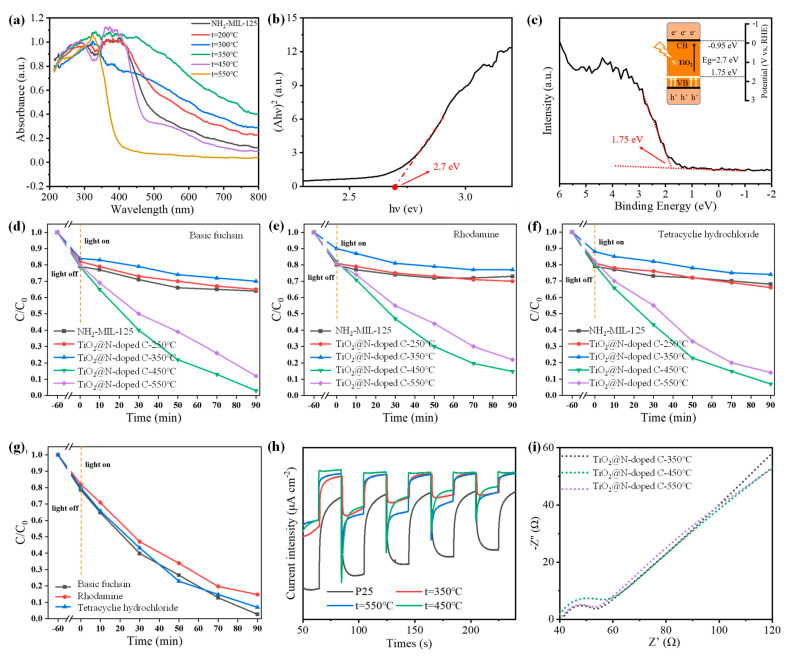
(**a**) DRS spectra for N-doped TiO_2_@C with calcination temperatures of 200 °C, 300 °C, 350 °C, 450 °C and 550 °C and for NH_2_-MIL-125. (**b**) Band gap and (**c**) valence band spectra of N-doped TiO_2_@C-450 °C (with an energy level diagram of N-doped TiO_2_@C-450 °C). Photocatalytic degradation efficiencies of (**d**) basic fuchsins, (**e**) Rhodamine B and (**f**) tetracycline hydrochloride over NH_2_-MIL-125, N-doped TiO_2_@C-200 °C, N-doped TiO_2_@C-350 °C, N-doped TiO_2_@C-450 °C and N-doped TiO_2_@C-550 °C under xenon lamp irradiation. (**g**) Degradation plots of N-doped TiO_2_@C-450 °C. (**h**) Transient photocurrent responses for N-doped TiO_2_@C with calcination temperatures of 350 °C, 450 °C and 550 °C and for NH2-MIL-125. (**i**) EIS Nyquist plots of N-doped TiO_2_@C with calcination temperatures of 350 °C, 450 °C and 550 °C.

The light absorption capacity of N-doped TiO_2_@C composites was investigated via a UV–Vis absorption test (Figure 3a). NH_2_-MIL-125 was yellow, as already described. The sample’s color continued to deepen as the calcination temperature rose due to the breakdown of the crystal skeleton and the collapse of the porous structure. The sample’s hue changed to brown at 350 °C, when it exhibited the best ability to absorb visible light. As the calcination temperature increased after 350 °C, TiO_2_ crystals started to form, and the sample’s color lightened. The sample was nearly white at 550 °C, and visible light could no longer be absorbed. We know that P25, the white commercial-grade TiO_2_, has poor absorption capabilities for visible light. The optimal catalyst for the best visible light absorption performance at 400–800 nm should be N-doped TiO_2_@C-350 °C. However, the photocatalytic activity of N-doped TiO_2_@C-350 °C was subpar due to the absence of TiO_2_ crystals. The most acceptable visible light catalyst, according to a thorough comparison between the sample’s photoabsorption capacity and TiO_2_ content, was N-doped TiO_2_@C-450 °C.

Compared with P25, the as-prepared N-doped TiO_2_@C-450 °C sample had a wider absorption range, which was because nitrogen in the organic connector was released and doped into the sample during calcination, which reduced the wide band gap of TiO_2_ and caused the light absorption range to shift to the visible region. The prohibited band spectra (Figure 3b) and valence band spectra (Figure 3c) of N-doped TiO_2_@C-450 °C can be used to compute the energies of the valence band, conduction band and band gap, which were −0.95 eV, 1.75 eV and 2.7 eV, respectively. The N-doped TiO_2_@C0-450 °C band gap created in this work was narrower than that of anatase TiO_2_, which had a band gap energy of 3.22 eV, making it more suitable for light absorption and photocatalytic processes.

Under xenon lamp illumination, the photocatalytic activity of N-doped TiO_2_@C composites was assessed using dealkalized fuchsin, Rhodamine B and tetracycline hydrochloride. These three contaminants all degraded in a very similar way. Only NH_2_-MIL-125 was capable of decomposing basic fuchsin (Figure 3d), lowering the pollutant concentration to 65%. This was because NH_2_-MIL-125 can carry out a certain amount of physical adsorption due to its large specific surface area and micropore structure. The capability for deterioration varied significantly among N-doped TiO_2_@C samples. At 350 °C, N-doped TiO_2_@C exhibited the lowest degradation ability compared to other catalysts. N-doped TiO_2_@C-350 °C showed the worst degradation ability, and 70% of basic fuchsin remained in the solution after degradation, even less than that of NH_2_-MIL-125’s adsorption effect. This was mainly due to two reasons. First, TiO_2_ does not form at this temperature and prevents photocatalytic reactions; second, at 350 °C, NH_2_-MIL-125 undergoes significant pyrolysis that rapidly changes its skeletal structure and reduces specific surface area sharply. Moreover, there was an overall increase in pore size, resulting in mesoporous pores and causing a decline in its adsorption effectiveness. Degradation of 99.7% of basic fuchsin with N-doped TiO2@C-450 °C was the highest among all samples within 90 min. However, anatase TiO_2_ obtained via the high-temperature heat treatment at 550 °C had the highest content and the best crystal form. However, the above characterization results indicate that the high-temperature heat treatment was not conducive to nitrogen doping and reduced the utilization efficiency of visible light. Consequently, the degradation efficiency of basic fuchsin was not as good as that achieved with N-doped TiO_2_@C-450 °C.

The degradation of Rhodamine B and tetracycline hydrochloride exhibited similar trends in the above samples (Figure 3e,f). NH_2_-MIL-125 achieved a 73% reduction in the content of Rhodamine B through physical adsorption. Among the tested catalysts, N-doped TiO_2_@C-350 °C demonstrated the lowest degradation efficiency with only 23% of pollutants being degraded, whereas N-doped TiO_2_@C-450 °C exhibited the highest degradation effect by degrading 89.9% of Rhodamine B within 90 min. Physical adsorption by NH_2_-MIL-125 resulted in a decrease in the content of tetracycline hydrochloride to 68%. Similarly, N-doped TiO_2_@C-350 °C showed inferior performance with only a 26% degradation rate for pollutants, whereas N-doped TiO_2_@C-450 °C displayed superior degradation capability by achieving a remarkable removal rate of 93% for tetracycline hydrochloride within the same time frame (Figure 3g). In Table 2, we summarize the recent works on the degradation performance of TiO_2_ composites against tetracycline. In comparison, our materials show outstanding catalytic properties [26].

Under xenon lamp irradiation, we used a photocurrent test to investigate the phenomenon of photogenerated carrier transport in samples. The photocurrent intensity of all N-doped TiO_2_@C samples was relatively high, indicating a slow photogenerated carrier recombination rate (Figure 3h). This was mainly because TiO_2_ was combined with the carbon matrix, and the porous structure of the carbon skeleton shortened the carrier transfer distance so that the photogenerated electron pairs could be effectively separated. In addition, the large specific surface area of the carbon skeleton exposed more active sites, making carrier transmission and diffusion more convenient. In short, the combination of carbon skeletons and TiO_2_ enhanced the photocurrent intensity of the composite compared with commercial-grade TiO_2_ P25. It is well known that the higher the photocurrent density, the better the ability to separate photogenerated electrons and holes. Therefore, compared with P25, N-doped TiO_2_@C improves carrier separation efficiency and inhibits photogenerated carrier recombination. To confirm this, we further studied the resistance of interfacial charge conversion via electrochemical impedance spectroscopy. As shown in Figure 3i, the radii of all N-doped TiO_2_@C composites were small, indicating that the N-doped TiO_2_@C catalyst had higher interfacial charge separation efficiency and better electrical conductivity, which is similar to the trend in the photocurrent response test results. The charge transfer resistance (Rct) of N-doped TiO_2_@C in an equivalent circuit was smaller than that of pure TiO_2_ [36], which further confirms the favored interfacial charge transfer.

In summary, the optimal balance between anatase TiO_2_ formation and nitrogen doping was achieved when the calcination temperature reached 450 °C, and N-doped TiO_2_@C-450 °C exhibited the highest photocatalytic capacity.

**Figure 4:**
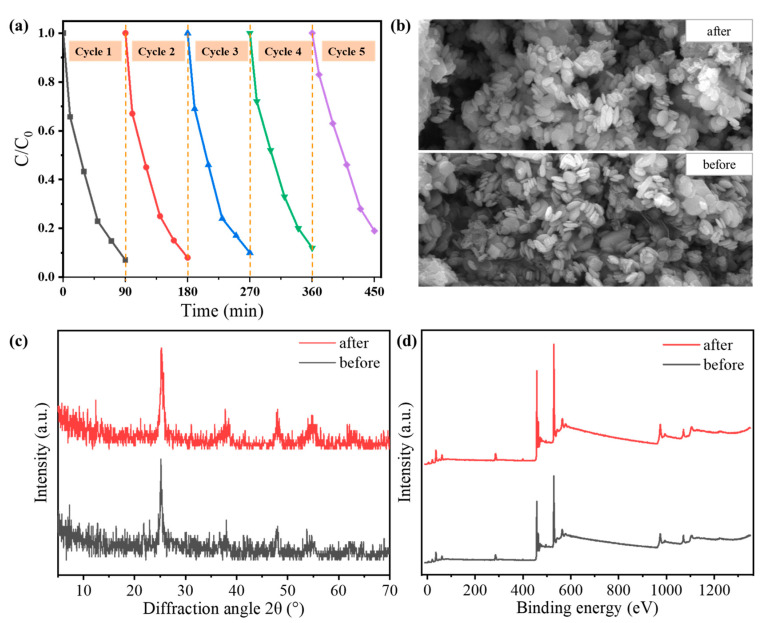
(**a**) Repeated experiments of tetracycline photodegradation over N-doped TiO_2_@C-450 °C. (**b**) SEM images, (**c**) XRD patterns and (**d**) XPS spectra of N-doped TiO_2_@C-450 °C before and after the photocatalytic reaction.

Tetracycline hydrochloride was used as an example to investigate the stability and reusability of the N-doped TiO_2_@C-450 °C catalyst. The same sample was employed for five consecutive degradation cycles under identical conditions (Figure 4a). No significant changes were observed after five cycles, although the degradation capacity decreased from 93% to 81%. This decrease in degradation capacity was primarily attributed to the blockage of mesoporous channels in N-doped TiO_2_@C-450 °C by pollutants over prolonged reaction times, making it challenging to effectively clean and negatively impacting the recyclability of the catalyst. Additionally, the inevitable loss of the photocatalyst during repeated use also resulted in a decrease in degradation efficiency. Moreover, a comparison of SEM images, XRD spectra and XPS spectra (Figure 4b–d) before and after the reaction revealed that there was no significant change in the structure, morphology characteristics and chemical composition of N-doped TiO_2_@C-450 °C before and after testing. N-doped TiO_2_@C-450 °C exhibited excellent photocatalytic ability, stability and reusability; it has great potential as a visible photocatalyst for various types of pollutants.

### 3.3. Photocatalytic Mechanism

**Figure 5:**
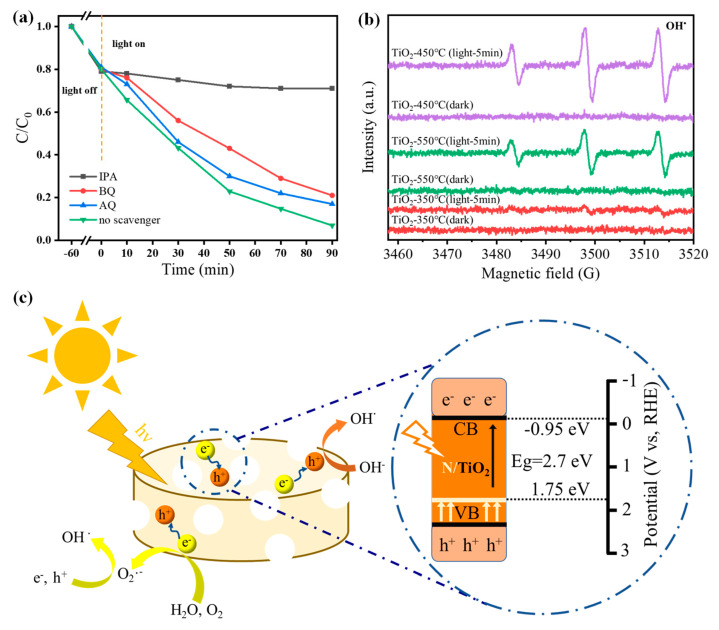
(**a**) Photocatalytic ability of N-doped TiO_2_@C-450 °C for the degradation of tetracycline with or without adding IPA, BQ and AO under visible light. (**b**) EPR spectra of OH• on N-doped TiO_2_@C-350 °C, N-doped TiO_2_@C-450 °C and N-doped TiO2@C-550 °C catalysts. (**c**) Probable photocatalytic mechanism of tetracycline degradation by N-doped TiO_2_@C-450 °C under visible light irradiation.

The photocatalytic mechanism of N-doped TiO_2_@C-450 °C nanomaterials during visible light degradation was investigated by conducting multiple trapping experiments using tetracycline hydrochloride as an example. Three additional trapping reagents were added to the reaction solution, among which ammonium oxalate (AO) was used to trap e^−^, benzoquinone (BQ) was used to trap O_2_•^−^ and isopropyl alcohol (IPA) was used to trap OH• (Figure 5a). The addition of each trapping agent affected the degradation rate of tetracycline hydrochloride, among which isopropyl alcohol had the most prominent inhibitory effect among all trapping agents. The degradation efficiency of N-doped TiO_2_@C-450 °C was reduced significantly to 71% after the addition of isopropyl alcohol, indicating that OH• plays a leading role in the degradation reaction of tetracycline hydrochloride under visible light irradiation. In addition, the addition of ammonium oxalate and benzoquinone (BQ) also had a certain effect on the degradation rate, suggesting that e^−^ and O_2_•^−^ were also involved in the process but did not play a major role. In conclusion, OH• was produced by the N-doped TiO_2_@C-450 °C catalyst during the photocatalysis process, which enhanced the oxidation capacity of the active substance and improved its ability to degrade tetracycline hydrochloride greatly.

A DMPO trapping agent was added to the photocatalytic reaction system to detect active free radicals via EPR. Therefore, electron paramagnetic resonance spectroscopy was used to detect the signals of OH• radicals generated by N-doped TiO_2_@C-350 °C, N-doped TiO_2_@C-450 °C and N-doped TiO_2_@C-550 °C under visible light irradiation (Figure 5b). Obviously, regardless of dark or light conditions, N-doped TiO_2_@C-350 °C OH showed almost no occurrence of free radicals, and this was because the TiO_2_ of N-doped TiO_2_@C-350 °C had not formed. For N-doped TiO_2_@C-450 °C and N-doped TiO_2_@C-550 °C, almost no OH• free radicals increased. This indicates that the photogenerated electrons on anatase TiO_2_ were transferred rapidly through the carbon skeleton, resulting in the production of a large number of OH• free radicals. N-doped TiO_2_@C-450 °C exhibited the highest number of OH• free radicals due to nitrogen doping, which enhanced the sample’s utilization rate of visible light. This phenomenon further confirms the excellent photocatalytic effect of nitrogen-doped TiO_2_ supported by a carbon skeleton, which can serve as photosensitizer to absorb visible light and help to promote charge carriers’ separation through cooperation with bulk/surface defects of TiO_2_ [37,38].

Based on the test results and analysis mentioned above, a possible photocatalytic reaction mechanism of N-doped TiO_2_@C-450 °C can be proposed (Figure 5c). Under visible light irradiation, nitrogen-doped TiO_2_ nanoparticles can efficiently produce charge carriers. At this stage, the unique porous structure of N-doped TiO_2_@C-450 °C provides additional pathways for the movement of charge carriers, facilitating the easy transfer of electrons to the carbon framework and effectively separating photogenerated electrons and holes. During the degradation process, O_2_•^−^ free radicals are generated through reactions between electrons enriched on the carbon framework with H_2_O and O_2_. It can be found that the REDOX potential of N-doped TiO_2_@C CB positions is more negative than that of O_2_/•O_2_^−^, and the REDOX potential of VB positions is more positive than that of OH^−^/•OH, thus contributing to the generation of reactive oxygen species under photoexcitation [39,40].

## 4. Conclusions

In summary, we successfully synthesized a N-doped TiO_2_@C composite material through NH_2_-MIL-125 template-assisted calcination. Supported by the NH_2_-MIL-125 carbon framework, the catalyst exhibited a significantly enhanced specific surface area of 63 m^2^/g, thereby facilitating an increased number of active sites for both the catalyst and reactant. The mesoporous structure (3.5 nm) of NH_2_-MIL-125 facilitated enhanced light penetration, thereby reducing the occurrence of light shielding. Element mapping confirmed the presence of nitrogen, indicating that nitrogen was released from the organic ligand 2-amino-terephthalic acid and doped into the sample during calcination. Nitrogen doping in anatase shortened its wide band gap of 3.22 eV to 2.7 eV, resulting in a shift in the optical absorption range toward the visible region. As a result, the N-doped TiO_2_@C catalyst exhibited extended optical absorption capabilities beyond just ultraviolet wavelengths. By carefully controlling the calcination temperature, we successfully synthesized various N-doped TiO_2_@C composites. When calcined at 450 °C, the N-doped TiO_2_@C catalyst achieved a balance between anatase content and nitrogen doping. The resulting N-doped TiO_2_@C-450 °C composite exhibited outstanding performance in photocatalytic degradation. Under visible light for 90 min, basic fuchsin, Rhodamine B and tetracycline hydrochloride were degraded by 99.7%, 89.9% and 93% respectively. Moreover, the N-doped TiO_2_@C-450 °C composite demonstrated excellent stability, as evidenced by negligible changes in SEM, XRD and XPS characteristics after five cycles of use; only a slight decrease in tetracycline hydrochloride degradation efficiency from 93% to 81% was observed. Last, we proposed the mechanism of photocatalytic degradation. This study presents a viable strategy for efficient pollution degradation under visible light.

## Figures and Tables

**Table 1:** Textural characteristics of NH_2_-MIL-125, N-doped TiO_2_@C-200 °C, N-doped TiO_2_@C-350 °C, N-doped TiO_2_@C-450 °C and N-doped TiO_2_@C-550 °C.

Sample	S_BET_ (m^2^g^−1^)	Vtotal (cm^3^g^−1^)	Average Pore Diameter (nm)	N/C *
NH_2_-MIL-125	539	0.087	5.32	0.146
N-doped TiO_2_@C-200 °C	763	0.168	6.54	0.0035
N-doped TiO_2_@C-350 °C	89	0.157	9.30	0.0022
N-doped TiO_2_@C-450 °C	63	0.141	10.89	0.0012
N-doped TiO_2_@C-550 °C	13	0.046	12.26	0.0007

* The N/C value means atomic mass ratio, which was taken with a Eurovector EA 3000 elemental analyzer in CHN mode.

**Table 2:** Summary of works on the degradation performance of TiO_2_ composites against tetracycline.

Order	Catalyst	Light Source	Concentrations of Catalysts (g/L)	Concentrations of Pollutants (mg/L)	Degradation Efficiency	Time (h)	Reference
1	g-C_3_N_4_/TiO_2_	300 Xe (UV–Vis)	0.4	20	90.1%	1.0	[26]
2	TiO_2_/Fe-MOF (15%)	300 Xe (UV–Vis, λ = 370 nm)	1	96	97%	4	[27]
3	TiO_2_, H_2_Ti_3_O_7_	Xe lamp	0.02	20	89%, 94%	1	[28]
4	Biofilm-UCPs-TiO_2_	lamp (20 W) of 1800 Lux	1	40	82.1%	24	[29]
5	Black-TiO_2_	SPD-16 UV–vis detector at 357 nm	0.5	10	66.2%	4.5	[30]
6	AgBreTiO_2_-Pal (50%)	200–800 nm by UV-Vis DRS	0.5	10	89.6%	1.5	[31]
7	Defect-rich hydrogenated g-C_3_N_4_/TiO_2_	300 Xe (λ > 400 nm)	0.6	30	60%	1.5	[32]
8	N-TiO_2_/Ov carbon nitride doped with oxygen	300 Xe (*λ* > 420 nm)	0.4	30	79.9%	1.0	[33]
9	Oxygen vacancies modified TiO_2_/O-terminated Ti_3_C_2_ composites	Vis (300 W)	0.4	20	88.5%	1.5	[34]
10	Ti_3_C_2_@TiO_2_	125 W Xe (*λ* > 400 nm)	1	20	90%	1.5	[35]
11	N-doped TiO_2_@C	300 W Xe (*λ* > 420 nm)	0.67	30	93%	1.5	This work

## Data Availability

The data presented in this study are available on request from the corresponding author.
